# ASSESSMENT OF SATISFACTION IN PATIENTS UNDERGOING SURGICAL TREATMENT BY THE WALANT TECHNIQUE

**DOI:** 10.1590/1413-785220243206e282517

**Published:** 2025-01-10

**Authors:** Marina Rafaele Makishi, Rafaela Amoedo Cox, Victor Spirandelli Pimentel, Yussef Ali Abdouni, Marcel Eiji Nakagawa

**Affiliations:** 1. Universidade Estadual de Campinas UNICAMP, Campinas, SP, Brazil.; 2. Santa Casa de Misericórdia de Sao Paulo, Pavilhao “Fernandinho Simonsen”, Programa de Residencia em Ortopedia e Traumatologia, Sao Paulo, SP, Brazil.; 3. Santa Casa de Misericórdia de Sao Paulo, Pavilhão “Fernandinho Simonsen”, Especialização em Mao e Microcirurgia, Sao Paulo, SP, Brazil.; 4. Universidade Salvador UNIFACS, Salvador, BA, Brazil.; 5. Universidade de Taubate UNITAU, Taubate, SP, Brazil.; 6. Universidade de Sao Paulo, Faculdade de Medicina, Hospital das Clinicas HC-FMUSP, Departamento de Ortopedia e Traumatologia, Sao Paulo, SP, Brazil.; 7. Santa Casa de Misericórdia de Sao Paulo, Faculdade de Ciências Medicas, Departamento de Ortopedia e Traumatologia, Sao Paulo, SP, Brazil.; 8. Centro Universitário do Planalto Central Aparecido dos Santos, Santos, SP, Brazil.; 9. Hospital Ipiranga, Departamento de Ortopedia e Traumatologia, Sao Paulo, SP, Brazil.

**Keywords:** WALANT, Carpal Tunnel Syndrome, Trigger Finger Disorder, De Quervain Disease, Heidelberg Questionnaire, Surgical Treatment, WALANT, Síndrome do Túnel do Carpo, Dedo em Gatilho, Tenossinovite de De Quervain, Questionário Heidelberg, Tratamento Cirúrgico

## Abstract

Objective: To analyze the satisfaction of patients who underwent hand surgical treatment with the wide-awake local anesthesia no tourniquet (WALANT) anesthesia technique. Methods: This is a cross-sectional study on the satisfaction of patients who underwent hand surgical treatment with the WALANT technique. These patients were treated at the Hand and Microsurgery outpatient clinic of a public hospital from March 2020 to March 2022. They were assessed by the modified Heidelberg questionnaire. Results: The overall average of satisfaction totaled 3.27 (maximum value 4, SD 0.52, p-value 0.04), representing a good result. Patients’ profile characteristics showed no statistically significant differences. Conclusion: The efficiency of WALANT technique stemmed from the high satisfaction rate of patients undergoing minor surgeries, justifying its use as a routine option in outpatient surgeries in the public system. *Level of evidence III, Retrospective comparative study.*

## RESUMO

Objetivo: Analisar o grau de satisfação dos pacientes submetidos ao tratamento cirúrgico de afecções na mão com anestesia pela técnica de WALANT. Métodos: Estudo transversal sobre o grau de satisfação dos pacientes submetidos ao tratamento cirúrgico de afecções na mão com anestesia pela técnica de WALANT (Wide Awake Local Anesthesia No Tourniquet), no ambulatório de Mão e Microcirurgia de um hospital público, durante os meses de março de 2020 a março de 2022, através da aplicação do questionário modificado de Heidelberg. Resultados: A média geral de satisfação foi de 3,27 (valor máximo 4, DP 0,52, p-valor 0,04), representando um bom resultado; não houve diferenças estatisticamente significantes em relação as características do perfil dos pacientes. Conclusão: Demonstrou-se a eficiência do uso da técnica de WALANT através do alto índice de satisfação dos pacientes submetidos a cirurgias de pequeno porte, justificando seu emprego como opção de rotina em cirurgias ambulatoriais no sistema público. *Nível de evidência III, Estudo retrospectivo comparativo.*

## INTRODUCTION

 The wide-awake local anesthesia on the tourniquet (WALANT) technique has been gaining popularity in hand surgery due to its various benefits: lower cost, shorter length of hospital stay, and greater ease and safety. It also enables the intraoperative evaluation if active movements. ^1^ ^,^ ^2^ The technique administers lidocaine, epinephrine, and sodium bicarbonate at the site to be operated, causing less bleeding and risk than the well-known Bier block and less discomfort by dispensing with tourniquets. ^3^ ^,^ ^4^ Moreover, WALANT causes no side effects such as nausea, vomiting, and urinary retention. ^3^ ^,^ ^5^

 Minor hand surgeries with the WALANT technique have a lower risk of anesthesia-related postoperative complications and offer greater safety for patients with comorbidities. ^1^ ^,^ ^5^ Also, surgical procedures with local anesthesia cost less than those using sedation, enabling a greater number of procedures in public services. ^1^ ^,^ ^3^ Several authors have evaluated hand surgeries by comparing anesthesia with sedation and the WALANT technique. Despite similar results, ^6^ WALANT showed a shorter post-anesthetic recovery of up to 60%. ^7^ In view of the difficulties scheduling this type of surgery in the Brazilian Unified Health System, the WALANT technique is an interesting option, allowing these procedures to be performed in a day hospital regime; especially in high-complexity hospitals, in which emergencies suspend elective surgeries and occupy beds for a long time. 

 Most of the literature has only informed the cost, efficacy, and fewer complications of the WALANT anesthetic technique, ignoring patients’ experiences. The complete evaluation of surgeries should include their satisfaction, to be determined based on the congruence between expected and obtained results. ^8^ ^,^ ^9^ This evaluation is difficult to obtain, ^9^ and several questionnaires and scores have been developed. Schiff et al. ^10^ developed and validated a multidimensional questionnaire — “The Heidelberg Peri-anaesthetic Questionnaire” — so anesthesiologists can find the causes of patients’ dissatisfaction. In turn, Moura et al. ^11^ validated this questionnaire for the Portuguese language, enabling the application of its questions and the evaluation of patients’ satisfaction toward the anesthetic procedures. 

This study applied a modified Heidelberg questionnaire to evaluate the degree of satisfaction of patients who had been subjected to surgical treatment for minor hand surgeries by the WALANT technique to reiterate and encourage its use as a routine option in outpatient surgeries at the Brazilian Unified Health System.

## MATERIALS AND METHODS

A cross-sectional study was carried out with the modified Heidelberg questionnaire in patients undergoing minor surgical treatment for hand conditions under the WALANT technique who had been followed at the Hand and Microsurgery Outpatient Clinic of a public hospital in São Paulo from March 2020 to March 2022. The questionnaire was applied by three resident physicians at the service during the consultations following the surgeries. This study was approved by the Ethics and Research Committee of the service under CAAE no. 58634822.5.0000.5479.

Surgical treatments for carpal tunnel syndrome (open decompression of the carpal tunnel), trigger finger (open A1 pulley release), and De Quervain tenosynovitis (opening of the first extensor compartment) were included as minor surgeries. A total of 63 patients were selected, of which, 30 answered the questionnaire and were included in this study.

The questionnaire is shown in Annex 1. The answers followed a scale of agreement scored in 4 items (4, totally agree; 3, agree; 2, disagree; 1, totally disagree). Characteristics of patients’ profile (such as age, gender and level of education) and their history of previous surgeries and type of anesthesia were also identified. Participants were also asked about details of their surgeries, fear of the procedure, waiting time, pain, surgery environment, and medical team availability.

### Inclusion criteria

- Patients of any gender aged over 18 years;
- Patients with trigger finger on any finger or hand, regardless of degree according to Green’s classification, ^2^ underwent A1 open pulley release under anesthesia by the WALANT technique without sedation;
- Patients with carpal tunnel syndrome of any laterality, regardless of the time since diagnosis, who had been subjected to open surgical treatment under anesthesia by the WALANT technique without sedation;
- Patients with De Quervain tenosynovitis of any laterality, regardless of the time since diagnosis, who had been subjected to open surgical treatment under anesthesia by the WALANT technique without sedation;
- Adequate post-surgical follow-up.

### Exclusion criteria

- Patients undergoing surgery with a technique other than WALANT;
- Patients undergoing surgery under sedation;
- Patients with diagnoses other than carpal tunnel syndrome, De Quervain tenosynovitis, or trigger finger;
- Refusal to participate in this study or unwillingness to answer the questionnaire.

### Data analysis

 The collected data were organized into tables on Microsoft Excel Office 2016 ^®^ , and statistically correlated by the Mann-Whitney and two-proportion equality tests and by the Spearman’s correlation on SPSS V20, Minitab 16, and Excel Office 2010. Statistical significance was set at p <0.05. 

 To facilitate statistical analysis, the scores of negative answers were reversed (the answer scored at “1” was converted to “4,” “2” to “3,” and so on) and marked with an “X.” Positive answers kept their score as the original given by the interviewee. The following questions had their scores reversed: 2, 3, 4, 6, 7, 10, 11, 12, 13, 14, 15, and 16 (Annex 1). 

## RESULTS

 Participants’ mean age totaled 57.6 years (SD 13.7 years, range 20-82), with 26 female and four male patients; 16 of which received a diagnosis of trigger finger; 12, of carpal tunnel syndrome; and two, of De Quervain tenosynovitis ( Table 1 ). 

 Asked about previous anesthesia, most patients (28 out of 30) had undergone a procedure with anesthesia, 21 reported lumbar plexus block (or spinal anesthesia); 18, having undergone general anesthesia and only seven, local anesthesia without sedation ( Table 1 ). 

 More than 50% had an educational level below complete elementary school (19) and only seven had higher education. The performed Spearman’s correlation found no statistically significant differences regarding participants’ answers ( Table 2 ). It ranges from −1 to 1. A positive correlation would mean that as the value an analyzed variable increases, so does that of the other and vice versa. Thus, no answer to the questions show a positive or negative correlation with education. 

Each question enables the evaluation of the average of the obtained answers, obtaining the degree of satisfaction; for which the closer the value to four, the greater the patient satisfaction.

**Figure 1.**
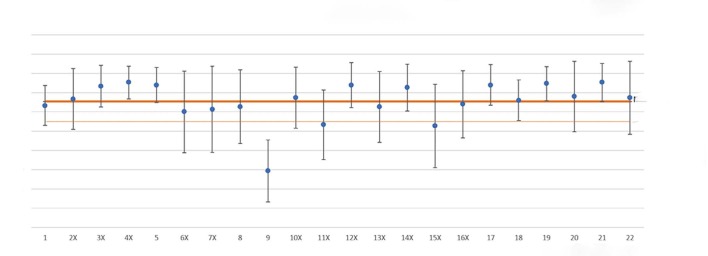
Mean, standard deviation, confidence interval in each question.

 Overall mean satisfaction totaled 3.27, with a 0.52 SD (p-value = 0.04). Of all analyzed questions, only question 9 stayed below the mean (evincing a lower satisfaction index), the mean of which equaled 1.47 and SD, 0.81. ( Figure 1 ) 

**Table 1.** Patient profile

Patient profile (n = 30)	
Variables	Values
Age (years) (mean±SD)	57.6 ± 13.7
Women	26 (86.7%)
Escolaridade	
Illiterate or Incomplete Elementary Education	15(50%)
Complete elementary school	4 (13.3%)
Complete secondary education	4 (13.3%)
Complete or incomplete higher education	7 (23.3%)
Prior anesthesia (yes)	28 (93.3%)
**Previous anaesthesia (n = 28)**	
Type of anesthesia	
General	18 (60%)
Spinal anesthesia	21 (70%)
Local	7 (23.3%)
Indication for surgical treatment	
Diagnosis	
Trigger finger	16 (53.3%)
Carpal tunnel syndrome	12 (40%)
De Quervain tenosynovitis	2 (6.7%)

Absolute values (n) and respective percentages (%).

Source: Medical archives of the institution

Questions 11 and 15 had their overall mean below the mean minus one standard deviation but obtained a satisfaction variance within the range of the overall mean. Question 11 refers to the waiting on the day of surgery (between hospitalization and referral to the operating room). This item obtained a 2.67 mean satisfaction and a 0.91 SD. Question 15 refers to patients shivering or feeling cold, showing a 2.63 mean satisfaction and a 1.08 SD.

**Table 2.** Spearman’s correlation and patients’ education

Correlation between education and obtained answers		
Question	Correlation	p-value
1	0.002	0.992
2	0.23	0.222
3	0.123	0.517
4	0.049	0.797
5	0.023	0.905
6	0.273	0.144
7	0.08	0.674
8	0.011	0.955
9	−0.232	0.217
10	−0.077	0.686
11	0.306	0.101
12	0.253	0.178
13	0.083	0.662
14	0.208	0.27
15	0.053	0.781
16	0.226	0.23
17	0.096	0.616
18	0.084	0.659
19	−0.136	0.473
20	0.03	0.873
21	0.018	0.926
22	0.04	0.832

Source: Medical archives of the institution

## DISCUSSION

 The complex evaluation of patients’ satisfaction involves several socioeconomic and cultural factors, previous personal experiences, and the surgical and anesthetic act itself. The literature currently considers satisfaction as a new indicator of health care quality, correlating it with treatment adherence and successful outcomes. ^9^ ^,^ ^12^ ^,^ ^13^ Thus, this study applied a modified “Heidelberg Peri-anaesthetic Questionnaire” that has been validated and translated into Portuguese. ^10^ ^,^ ^11^ The original Heidelberg questionnaire was adapted for performance by surgeons rather than anesthesiologists, excluding questions that could become repetitive. 

 Furthermore, the original model Schiff ^10^ proposed distributed the questionnaire for filling by patients. This study applied this questionnaire by interviews to reduce biases in filling out the questionnaire and lack of answers and increase research adherence. ^8^ ^,^ ^14^ However, interviews could suffer the influence of the interviewer’s (the surgeon) presence as a bias, even if involuntary. ^8^ To minimize this, the interviewer differed from the physician who performed the patient’s surgery in this study. 

 Schiff et al. ^10^ found higher levels of satisfaction in older patients and in lower levels of education. Myles et al. ^15^ and Heidegger et al. ^16^ reported lower satisfaction in younger interviewees. Moura et al. ^11^ and Lemos ^8^ reported lower satisfaction rates the higher the level of education. In total, 43% of the patients in this study were aged over 60 years, with a mean age of 57.6 years; 50% of whom had poor education (incomplete elementary school, below nine years of schooling). Overall average satisfaction was high — 3.27 (p-value 0.04), as in other studies, despite no statistically significant correlation between satisfaction indices and education or age. 

 Previous studies reported lower satisfaction rates regarding fear of anesthesia; anxiety waiting for surgery; and pain, thirst, and drowsiness. ^8^ ^,^ ^10^ ^,^ ^14^ In this study, question 11 (“the waiting time on the day of surgery was long”) showed a mean satisfaction below the general average, with an index of 2.67 (SD 0.91) versus 3.27 (SD 0.52; p-value 0.04), which, although not as high as in other studies, proved to be a parameter with the worst evaluation. Several authors have shown that the organization of a health system also influences satisfaction indices as prolonged waiting increases anxiety, agitation, and discontent. ^8^ ^,^ ^17^ ^,^ ^18^ Thus, organizational systems should undergo reforms to reduce waiting and thus anxiety and improve the quality of the provided services. 

 Question 15 also showed a lower index than the other parameters, with a mean of 2.63 (SD 1.08). Other studies have mentioned no unsatisfactory evaluations. ^8^ ^,^ ^10^ ^,^ ^11^ ^,^ ^14^ This item asked if patients “felt cold or shivered in the room in which they were anesthetized.” Thus, better temperature control in operating rooms can improve patient comfort during procedures. 

 On the other hand, analyzing fear of anesthesia or surgery (questions 6 and 7) obtained no dissatisfaction rates, showing high averages — 3.0 (SD 1.06) and 3.07 (SD 1.12), respectively —, unlike previous studies. Thus, the WALANT technique can be considered comfortable. Pain, thirst, or drowsiness also obtained no dissatisfaction rates in this study, receiving mentions in previous studies. ^8^ ^,^ ^10^ ^,^ ^11^ ^,^ ^14^ The exclusive application of the WALANT technique dispensed with sedation or any other anesthesia, prolonged fasting, or sedatives. Thus, patients showed no such complaints, reinforcing the comfort that the WALANT technique provides in minor procedures. 

 Moura et al. ^11^ found higher rates of dissatisfaction in women. The 86.7% prevalence of female patients in this study (p-value< 0,001) showed no correlation with worse indices. The predominance of females in this study stems from the fact that the main diseases the WALANT technique treat occur more prevalently in women, who encompass 80% of the cases of carpal tunnel syndrome ^19^ and up to 95% of those of De Quervain tenosynovitis. ^20^ Other analyzed factors that could have negatively influenced outcomes included previous anesthesia and local anesthesia. In total, 93.3% of patients (p-value < 0.001) reported previous anesthesia and 76.7%, (p-value < 0.001) local anesthesia. However, despite most patients having previous knowledge, this failed to obtain an unsatisfactory evaluation of the WALANT technique. On the contrary, the overall average showed satisfaction and approved its use. 

 The lowest positive index this study obtained refers to question 9 — “the surgery was postponed to another day”, with a mean of 1.47 (SD 0.81). Since the surgery of most patients showed no postponement, this question steered away from the final agreement standard, differing from the general satisfaction average. Rather than reflecting negatively on the service, it exemplifies its commitment and organization. Moreover, the low rate of postponement of surgeries evinces the ease, greater applicability, and lower risks related to anesthesia despite comorbidities. ^3^ ^,^ ^5^ ^,^ ^21^

The questions 4,5,12,17,19, and 21 showed the highest satisfaction rates. Questions 4 and 5 refer to explanations given in the consultation prior to surgery, showing satisfaction rates of 3.77 (SD 0.42) and 3.70 (SD 0.46), respectively. Question 17 — “anesthesia took place exactly as the physician had explained” — had a 3.70 satisfaction index (SD 0.53), i.e., patients easily understood the entire process of anesthesia with WALANT. Questions 19 and 21 show patient satisfaction toward the care and assistance from the surgical team, with indices equal to 3.70 (SD 0.44) and 3.77 (SD 0.50), showing that the satisfactory result of the entire process also involves the intraoperative care offered to patients. Thus, providing clear and easy-to-understand information and good care during surgery will influence good results and satisfaction.

 Item 12 — “feeling alone bothered you” — obtained a good satisfaction index of 3.70 (SD 0.59), differing from the results in Schiff et al. ^10^ as the patients in this study reported no such discomfort. The service of this study performed the surgeries in a day hospital, in which patients stayed with their companion up to the moment of entering the operating room, reuniting with them soon after the procedure. The positivity of this evaluation reiterates good indicators for the WALANT technique. 

 Some limitations in this study stem from its relatively small (n=30) and predominantly female and poorly educated sample and the application of the questionnaire by interview. Moreover, standardization only included surgeries to treat the three chosen conditions, limiting the demonstration of the WALANT technique. This study also ignored cost-benefit since previous studies had shown it. ^1^ ^,^ ^7^ ^,^ ^22^ A future alternative would include expanding research by analyzing more patients with other diagnoses and hospital costs from the WALANT technique without sedation in comparison to other anesthestic methods. 

However, the modified questionnaire usefully assessed the satisfaction of patients subjected to the WALANT technique, with satisfactory results in favor of its use.

## CONCLUSION

Several countries perform minor hand surgeries with the WALANT technique (the patient awake and without sedation) increasingly more often. It shows proven safety, efficacy, and accessibility.

This study complemented the efficiency of the WALANT technique by showing the high level of satisfaction of patients operated with this anesthetic technique, justifying its use in outpatient surgeries within the public system.

Moreover, evaluating patient satisfaction goes beyond analyzing surgical results: analgesia, welcoming, and perception of the quality of service and care should also receive prioritization.

## Annex 1. Questionário de Heidelberg modificado

**A. Identificação do paciente:**

- Sexo
- Idade
- Escolaridade
  - Analfabeto ou fundamental incompleto
  - Fundamental completo
  - Ensino médio completo
  - Superior incompleto ou completo
- Anestesias anteriores

**B. Questionário:**

(Avaliação: 1- Discordo totalmente; 2 – discordo; 3- concordo; 4- concordo totalmente)

1. Antes da cirurgia, o contato com o médico foi efetuado num ambiente agradável

2. O médico, que o contactou antes da cirurgia, deveria ser mais simpático

3. O médico, que o contactou antes da cirurgia, parecia estar com pressa

4. O médico, que o contactou antes da cirurgia, não deu informação suficiente

5. A informação dada pelo médico, que o contactou antes da cirurgia, foi fácil de entender

6. O medo da anestesia foi importante para si

7. O medo da cirurgia foi importante para si

8. Na noite antes da cirurgia sentiu-se calmo

9. A cirurgia foi adiada para outro dia

10. Antes da cirurgia sentiu um medo incontrolável

11. O tempo de espera no dia da cirurgia foi longo

12. Sentir-se sozinho/a incomodou-o/a.

13. O medo ou agitação no momento antes da anestesia foi importante

14. A sede antes da anestesia foi um problema para si

15. Sentiu frio ou tremor na sala onde foi anestesiado/a.

16. Dor antes da anestesia causou-lhe ansiedade.

17. A anestesia decorreu exatamente como o médico tinha explicado.

18. O ambiente na sala onde foi anestesiado/a era agradável.

19. Os membros da equipe cuidaram bem de si e foram prestáveis enquanto era anestesiado/a

20. Não teve dor nenhuma ou quase nenhuma noutras áreas do corpo após a cirurgia (por exemplo, cabeça).

21. Os membros da equipa mostraram que estavam verdadeiramente preocupados com a sua dor.

22. Os membros da equipe rapidamente aliviaram a sua dor.
